# Supplementary figures and images for: YOLOv8-DEE: a high-precision model for printed circuit board defect detection
Source: PeerJ Comput Sci. 2024 Dec 12;10:e2548. doi: 10.7717/peerj-cs.2548 (PMC11888845; doi:10.7717/peerj-cs.2548)

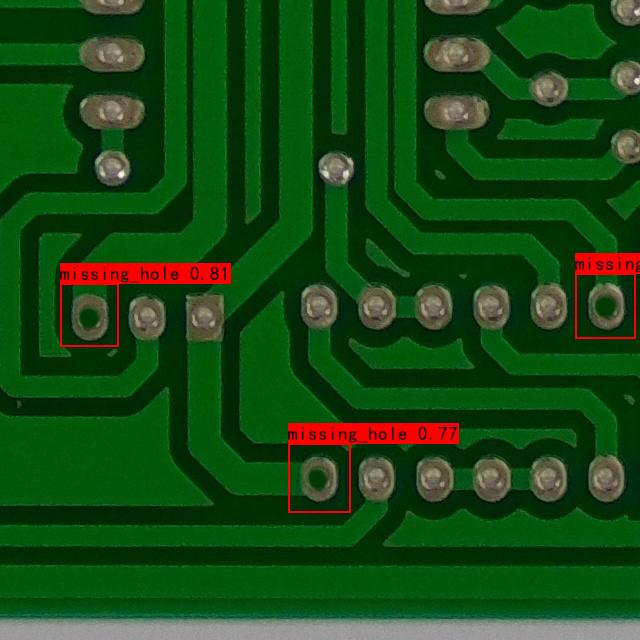

Supplement: Supplemental Information 1 [file peerj-cs-10-2548-s001.zip › img.jpg]

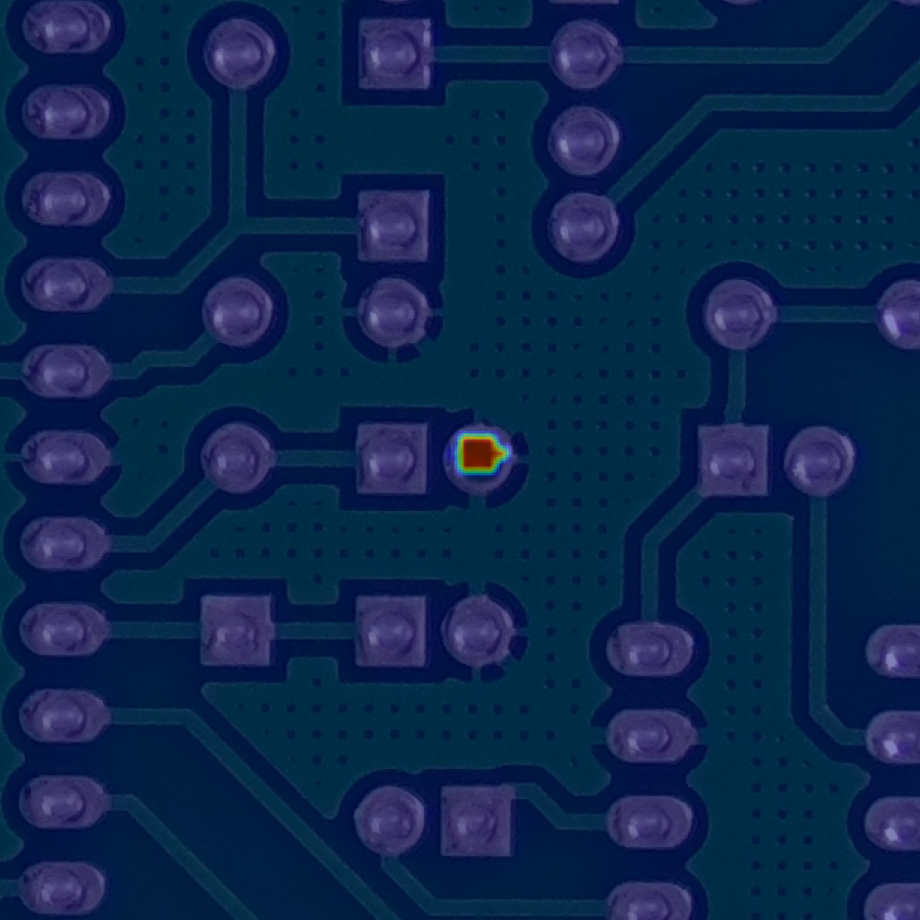

Supplement: Supplemental Information 1 [file peerj-cs-10-2548-s001.zip › model_data/heatmap_vision.png]
